# Serum Metabolomics Signatures Associated With Ankylosing Spondylitis and TNF Inhibitor Therapy

**DOI:** 10.3389/fimmu.2021.630791

**Published:** 2021-02-19

**Authors:** Jiayong Ou, Min Xiao, Yefei Huang, Liudan Tu, Zena Chen, Shuangyan Cao, Qiujing Wei, Jieruo Gu

**Affiliations:** Department of Rheumatology, The Third Affiliated Hospital of Sun Yat-sen University, Guangzhou, China

**Keywords:** ankylosing spondylitis, metabolomics (OMICS), TNF inhibitor, liquid chromatography-mass spectroscopy, biomarker

## Abstract

Ankylosing spondylitis (AS) is a type of spondyloarthropathies, the diagnosis of which is often delayed. The lack of early diagnosis tools often delays the institution of appropriate therapy. This study aimed to investigate the systemic metabolic shifts associated with AS and TNF inhibitors treatment. Additionally, we aimed to define reliable serum biomarkers for the diagnosis. We employed an untargeted technique, ultra-performance liquid chromatography-mass spectroscopy (LC-MS), to analyze the serum metabolome of 32 AS individuals before and after 24-week TNF inhibitors treatment, as well as 40 health controls (HCs). Multivariate and univariate statistical analyses were used to profile the differential metabolites associated with AS and TNF inhibitors. A diagnostic panel was established with the least absolute shrinkage and selection operator (LASSO). The pathway analysis was also conducted. A total of 55 significantly differential metabolites were detected. We generated a diagnostic panel comprising five metabolites (L-glutamate, arachidonic acid, L-phenylalanine, PC (18:1(9Z)/18:1(9Z)), 1-palmitoylglycerol), capable of distinguishing HCs from AS with a high AUC of 0.998, (95%CI: 0.992–1.000). TNF inhibitors treatment could restore the equilibrium of 21 metabolites. The most involved pathways in AS were amino acid biosynthesis, glycolysis, glutaminolysis, fatty acids biosynthesis and choline metabolism. This study characterized the serum metabolomics signatures of AS and TNF inhibitor therapy. We developed a five-metabolites-based panel serving as a diagnostic tool to separate patients from HCs. This serum metabolomics study yielded new knowledge about the AS pathogenesis and the systemic effects of TNF inhibitors.

## Introduction

Ankylosing spondylitis (AS) belongs in the group of diseases called spondyloarthropathies, presenting with chronic back pain, which predominantly affects the spine and the sacroiliac joints. AS is more common in males, presenting with inflammatory back pain, the onset of which typically occurs in the third or fourth decade of life. At the advanced stage, the disease progression may result in spinal deformity, limitations of spinal mobility and inevitably impaired quality of life. The etiology of AS remains unclear. According to studies in twins, genetic factors are now thought to account for over 90% of the risk for AS (1). Despite the numerous disease-associated variants identified in AS with genome-wide association studies, they cumulatively explain only a small proportion (<28%) of the heritability of these diseases (2). Environmental exposures have been suspected to play a role in AS, such as mechanical stress, infections (3), smoking (4), and breast feeding (5) in the early life.

Due to the insidious onset or ignorance of the lower back pain, the diagnosis for AS is often delayed by 5–10 years (6). The administration of biologics therapies, which has been proven highly effective in multiple clinical trials, could achieve a high rate of clinical remission. However, the lack of early diagnosis tools often delays the institution of appropriate therapy. Consequently, there is an unmet need for more effective and sensitive biomarkers of diagnosis. The introduction of tumor necrosis factor (TNF) inhibitors nearly two decades ago opened a new chapter of the treatment of AS, especially in patients with insufficient response to conventional treatment and inhibit radiographic progression (7). Nonetheless, the underlying pathophysiology targeted by anti-TNFα therapy has not yet been elucidated.

As one of the ‘omics’ technologies, metabolomics is a fast-developing research area in the post-genomic era. It has emerged to be a powerful comprehensive approach to characterize convoluted metabolic changes and evaluate the biochemical mechanisms involved in such changes in a systematic fashion (8). Currently, metabolomics has been utilized in biomarker discovery in multiple rheumatic diseases, including rheumatoid arthritis (RA) and reactive arthritis (9, 10). However, the application of metabolomics to AS is still in its infancy, although several studies have been reported recently (11–13). Overall, the sample size of most studies is small and the types of samples are diverse including plasma, fecal, and urine. The two most common detection methods in metabolomics, nuclear magnetic resonance and mass spectrometry, have not yet been employed, but the findings are contradictory among studies. Moreover, the follow-up data regarding metabolic alteration in patients treated with TNF inhibitors are scarce.

Therefore, this study aimed to investigate the systemic metabolic shifts associated with AS and define reliable serum biomarkers for the diagnosis of AS. Additionally, we aimed to further investigate the influence of 24-week TNF inhibitor treatment on metabolic profiles in AS. To accomplish these objectives, we employed an untargeted technique with high sensitivity and specificity, namely ultra-performance liquid chromatography-mass spectroscopy (LC-MS), to analyze the serum metabolome of 32 AS individuals and 40 healthy controls (HCs). Furthermore, we attempted to construct a metabolites-based diagnostic panel to distinguish AS from the healthy controls. Besides, metabolome profiles were also compared before and after treatment with TNF inhibitors. We propose that the serum metabolite signatures can assist in diagnosis and provide insight into the underlying pathophysiology of AS and the systemic effects of TNF inhibitors.

## Materials and Methods

### Study Participants

A total of 32 AS patients were enrolled in this study from the Rheumatology department in the Third Affiliated Hospital of Sun Yat-sen University between June 2016 and June 2018. Additional 40 sex- and age-matched HCs from the physical examination center in our hospital were consecutively recruited. Inclusion criteria of AS patients were as follows:1) aged over 16 years old; 2) fulfilled the modified 1984 New York diagnosis criteria; 3) patients did not take any TNF inhibitors treatment before enrollments; 4) active disease (Bath Ankylosing Spondylitis Disease Activity Index (BASDAI) ≥4.0 or Ankylosing Spondylitis Disease Activity Score-CRP (ASDAS-CRP) ≥1.3); 5) the patients administered TNF inhibitors (Etanercept) over 24 weeks. Patients with other rheumatic diseases, other systemic diseases or tumors were excluded from this study. Patients who took conventional disease-modifying antirheumatic drugs (DMARDs) or medicine impacting serum metabolites (such as insulin and statin) were also excluded. All HCs had no history of chronic disease or rheumatic diseases. Demographic and clinical parameters including age, sex, symptom duration, BASDAI, Bath Ankylosing Spondylitis Functional Index (BASFI), ASDAS-CRP, and laboratory indicators, such as HLA-B27, C-reactive protein (CRP) and erythrocyte sedimentation rate (ESR) were recorded. The serum samples of AS patients were collected before and after the 24-week TNF inhibitors treatment. All procedures involving human participants in the study were performed in accordance with the 1964 Helsinki declaration. The protocol was approved by the Ethics Committee of the Third affiliated Hospital, Sun Yat-Sen University ([2013]2-93). All patients signed informed consent prior to study enrollment.

### Sample Collection and Preparation

The detailed protocols of samples collection, preparation, metabolomics profiling and data pre-processing are available in **Supplementary Data**.

### Data Processing and Statistical Analysis

The data processing procedures comprised filtering, imputation of missing values (R package “DMwR”) and area normalization. Then the data matrix was imported into SIMCA-P software (version 14.1, Umetrics AB, Umea, Sweden) for multivariate statistical analysis including principal component analysis (PCA) and orthogonal partial least-squares discriminant analysis (OPLS-DA). Metabolites were further applied to the univariable Wilcoxon rank-sum test. The differential metabolites that satisfied the criterion of variable importance in the projection (VIP) values of >1.0 and false discovered rate (FDR) of <0.05 were considered as biomarker candidates. A diagnostic model was established with Least absolute shrinkage and selection operator (LASSO) regression (R package “glmnetcr”). The results were presented as mean ± standard deviation (SD) for continuous variables and as percentage for categorical variables. GraphPad Prism (version 6.02, San Diego, CA, USA) was used for statistical analysis of the data. *P* < 0.05 was considered statistically significant.

## Results

### Demographic Characteristics of the Study Population

**Figure 1:**
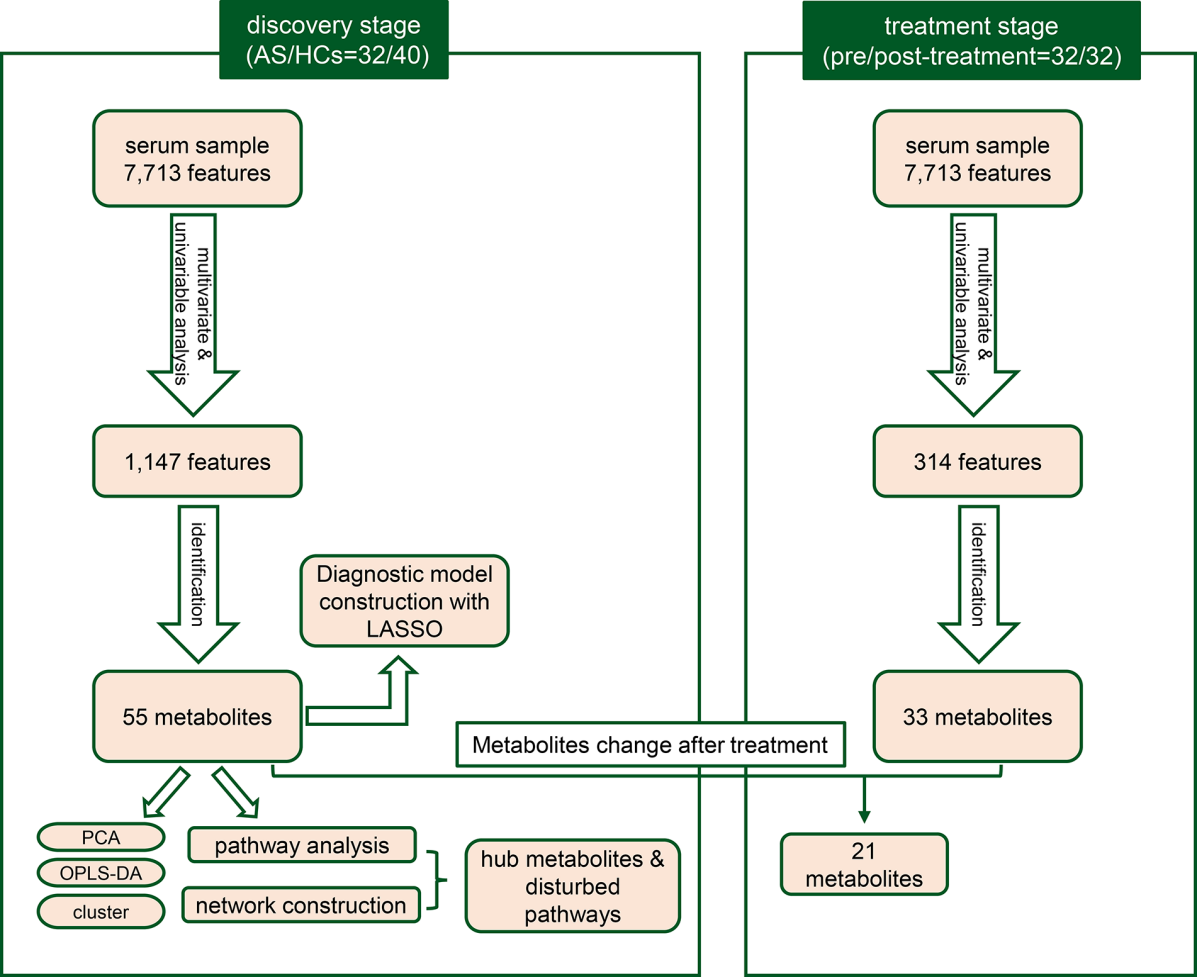
The workflow for the study design and analytical pipeline.

A total of 72 individuals (32 AS patients and 40 HCs) were enrolled in the serum metabolic profiling study. Flow diagram of the overview for the study design and analytical pipeline was depicted in **Figure 1**. All patients and HCs were included in the discovery stage. The treatment stage consisted of 32 follow-up patients who received 24-week TNF inhibitors therapy. The general demographic and clinical characteristics of the study participants were presented in **Table 1**. Age and gender were matched between AS patients and HCs. Most of the participants in the two groups were male (92.5% vs. 90.6%, *p*>0.05) and the mean ages were 27.05 ± 5.64 years vs. 28.63 ± 7.53 years (*p*>0.05). The mean disease duration of patients was 94.28 mouths. Indicators of clinical assessments significantly improved after treatment, while the acute phase reactants (ESR and CRP) significantly decreased (*p*<0.001 respectively). Besides, a significant reduction of BASDAI, BASFI, and ASDAS-CRP was observed (*p*<0.001 respectively). These results indicated the therapeutic benefit of TNF inhibitor treatment.

**Table 1:** The demographic characteristics of the study population.

Characteristics	HC (n = 40)	AS (pre-treatment) (n = 32)	AS (post-treatment) (n = 32)	*p*
Male, n (%)	37 (92.5)	29 (90.6)	–	1.000*
Age, year, mean ± SD	27.05 ± 5.64	28.63 ± 7.53	–	0.314*
HLA-B27 positive, n (%)	–	30 (93.8)	–	–
Duration, month, mean ± SD	–	94.28 ± 48.79	–	–
BASDAI, mean ± SD	–	6.85 ± 2.03	3.28 ± 1.70	<0.001
BASFI, mean ± SD	–	4.46 ± 2.22	2.37 ± 2.01	<0.001
ASDAS-CRP, mean ± SD	–	4.36 ± 0.79	1.07 ± 0.73	<0.001
ESR, mm/H, median (IQR)	–	29.5 (12.5–46.5)	5.0 (3.0–10.8)	<0.001
CRP, mg/L, median (IQR)	–	21.1 (12.4–44.0)	2.6 (1.1–9.5)	<0.001

*P-values were calculated with HCs as references. Other p-values were calculated from the comparison between pre- and post-treatment. HC, health control; AS, ankylosing spondylitis; SD, standard deviation; BASDAI, Bath Ankylosing Spondylitis Activity Index; BASFI, Bath Ankylosing Spondylitis Functional Index; ASDAS, Ankylosing Spondylitis Disease Activity Score; ESR, erythrocyte sedimentation rate; CRP, C-reactive protein; IQR, interquartile range.

### Metabolomic Characteristics of TNF Inhibitors-Naive AS Patients

**Figure 2:**
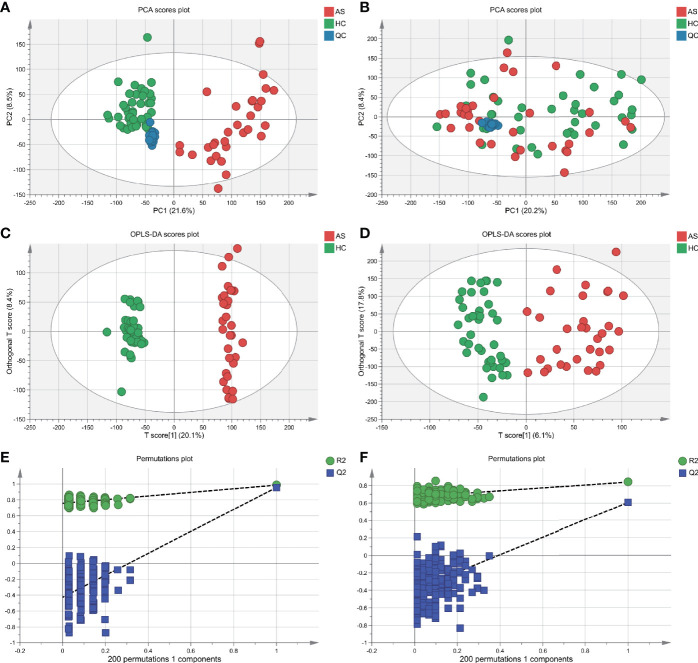
Multivariate statistical analysis of serum metabolites in ankylosing spondylitis patients (AS) and healthy controls (HCs). **(A, B)** The scatter plots of principal component analysis (PCA) were based on the serum metabolic data in positive and negative ion mode. AS patients and HCs were denoted with red and green circles, respectively. The QC samples (blue circles) were clustered together in two modes. **(C, D)** Orthogonal partial least-squares discriminant analysis (OPLS-DA) scatter plots were based on the serum metabolic profiles in positive and negative ion mode. **(E, F)** The statistical validation of the corresponding OPLS-DA models by permutation tests (200 times).

With ultra-performance LC-MS, a total of 7,713 metabolic peaks including 5,349 in positive ion mode and 2,364 in negative ion mode were detected from 72 subjects. The representative total ion chromatograms (TIC) for QC samples were overlapped almost completely. The RSD of QC samples in each metabolic peak was also used to assess the stability of the detection system, and 4,952 (92.6%) and 2,092 (88.5%) metabolic peaks with RSDs less than 30% were left in positive and negative ion mode respectively. Furthermore, PCA analysis was performed to determine the classification of samples from patients and control. As is shown in **Figures 2A, B**, there were no obvious outliers in the serum samples of two modes and the QC samples were clustered together. These results indicated the outstanding stability of the detection condition. Additionally, automated clear separation was observed in the positive mode rather than the negative mode in this unsupervised analysis. From the OPLS-DA models (**Figures 2C, D**), the AS group could be distinguished from HCs with good fitness (R2Y = 0.986, Q2 = 0.956 in positive ion mode; R2Y = 0.842, Q2 = 0.608 in negative ion mode). The cross-validation through permutations tests (200 times) of two OPLS-DA models validated that there was no overfitting of the models (generated intercepts of R2 = 0.769, Q2 = −0.408 and R2 = 0.665, Q2 = −0.433 respectively) (**Figures 2E, F**). As a result, a total of 1,278 metabolic peaks with VIP-values larger than 1.0 were considered for the subsequent analysis. Through the univariable Wilcoxon rank-sum test, altogether 1,147 metabolic peaks were found to be significantly altered between AS subjects and HCs (*p*<0.05). Finally, these peaks were mapped to 55 metabolites by database searches, in which 41 were elevated and 11 decreased. The metabolites consisted of amino acids, carbohydrates, fatty acyls, glycerophospholipids, purines and purine derivatives. The detailed information including HMDB ID, VIP-values and fold change of individual metabolites was presented in **Supplementary Tables S1, S2**.

**Figure 3:**
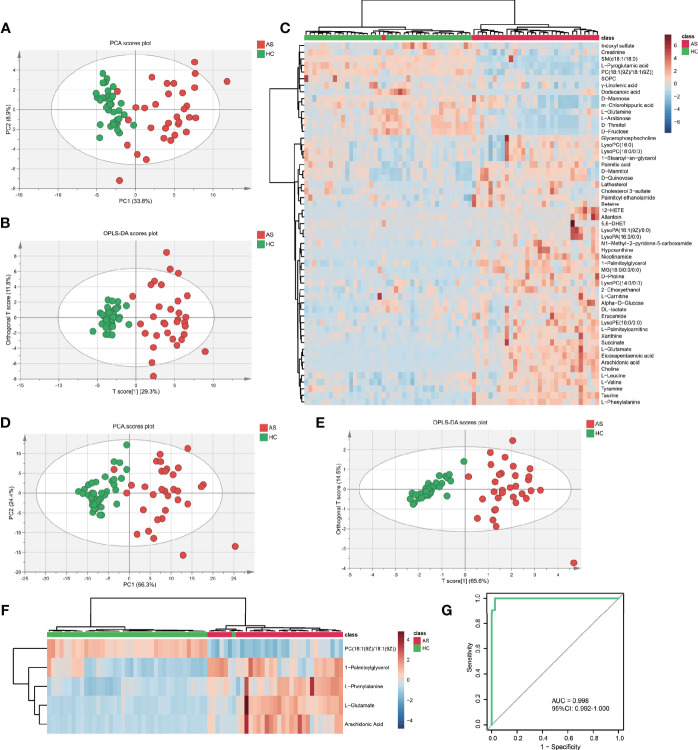
The capacity of 55 discrepant serum metabolites and LASSO-based model in discrimination. **(A)** The scatter plot of principal component analysis (PCA) was based on 55 metabolites to discriminate ankylosing spondylitis patients (AS, red circles) from healthy controls (HCs, green circles). **(B)** Orthogonal partial least-squares discriminant analysis (OPLS-DA) scatter plot of 55 metabolites. **(C)** Unsupervised cluster heatmap of 55 metabolites. **(D)** The scatter plot of PCA was based on 5 metabolites. **(E)** OPLS-DA scatter plot of 5 metabolites. **(F)** Unsupervised cluster heatmap of five metabolites. **(G)** Receiver operating characteristic curve (ROC) analysis of the 5 metabolites panel for diagnosis.

To further test the classification power of these 55 metabolites, we also performed unsupervised multivariate analysis. PCA analysis of the two groups, using the 55 unique metabolites, revealed distinct metabolomic profiles (**Figure 3A**). Furthermore, the OPLS-DA analysis enabled a clear separation between the AS group and HCs (**Figure 3B**). Unsupervised clustering analysis showed the 55 metabolites could distinguish most of the AS patients from HCs (**Figure 3C**). The ROC curve analyses were conducted based on three multivariate algorithms. The combination of these 55 metabolic peaks had AUC values of 0.861 (95% CI 0.696–0.969), 0.999 (95% CI 0.993–1) and 0.992 (0.959–1) in the PLS, random forest, and SVM prediction models respectively. These results suggested that these 55 differential metabolites could serve as candidate biomarkers in AS diagnosis, while more screening was needed.

### Establishment of Five-Metabolites-Based Diagnostic Model

To establish the least redundant and most informative diagnostic model for clinical application, we used a more advanced statistical tool, the LASSO procedure. Only 33 candidate metabolites with AUC greater than 0.75 were guided in the initial LASSO model. Finally, we obtained a five-metabolites-based diagnostic model, consisting of L-glutamate, arachidonic acid, L-phenylalanine, 1-palmitoylglycerol, PC (18:1(9Z)/18:1(9Z)). The PCA (**Figure 3D**) and OPLS-DA (**Figure 3E**) exhibited excellent discriminative power between AS patients and HCs. The unsupervised clustering analysis showed that the five metabolites can distinguish most AS patients from HCs (**Figure 3F**). Moreover, the AUC in ROC analysis was used to evaluate the diagnostic performance of the biomarker panel. The final model had a fairly high AUC value equal to 0.998 (95%CI: 0.992–1.000) (**Figure 3G**) and explained 63.8% of the variation in AS patients and controls. Taken together, these results demonstrated that five metabolites-panel could be considered a promising tool for diagnosis of AS.

### Metabolomic Alteration in Response to TNF Inhibitors Treatment

Subsequently, we examined how TNF inhibitors treatment affected the serum metabolites in the 32 AS patients. There are no obvious outliers, however, unlike the separation in AS vs. HCs, there is no apparent separation in pre vs. post-treatment samples in PCA (**Supplementary Figures S1A, B**). In OPLS-DA models, the post-treatment samples could be mostly distinguished from pre-treatment samples (**Supplementary Figures S1C, D**) with good discrimination and predictive ability (R2Y = 0.871, Q2 = 0.327 in positive ion mode; R2Y = 0.660, Q2 = 0. 176 in negative ion mode). It was verified that the models were not over-fitted in permutation tests (**Supplementary Figures S1E, F**). The metabolism of AS patients was altered after TNF inhibitors treatment, albeit not as conspicuous as the difference between AS patients and HCs. A total of 33 metabolites exhibited abundance shifts after the administration of TNF inhibitors treatment, with 10 upregulated metabolites and 21 downregulated metabolites. Among these significantly altered metabolites, 21 metabolites were significantly restored in the directions concordant with HCs. As observed in **Supplementary Figure S2A**, the abundance of 10 metabolites (including L-leucine, glycerophosphocholine, m-chlorohippuric acid, D-threitol, D-fructose, D-mannose, palmitic acid, cholesterol 3-sulfate, D-mannitol, D-quinovose) were not significantly different between HCs and post-treatment AS patients. However, 11 metabolites (taurine, L-glutamate, hypoxanthine, L-phenylalanine, nicotinamide, eicosapentaenoic acid, L-palmitoylcarnitine, SOPC, L-pyroglutamic acid, succinate, L-glutamine) remain significantly different (**Supplementary Figure S2B**). The results provided further evidence that TNF inhibitors treatment can reverse the aberrant metabolism state toward that of the controls, albeit in an insufficient way.

Furthermore, we aimed to identify potential metabolite biomarkers to predict response to TNF inhibitors. The patients were divided into TNF inhibitors sensitive (n=21) and resistant (n=11) groups according to the Assessment of Spondylarthritis international Society (ASAS) Response Criteria for a 20% improvement (ASAS20). However, the PCA did not show discrimination among the two groups and the OPLS-DA models were overfitted. The results suggested that serum metabolomics might not be able to predict responses to TNF inhibitors in AS patients.

### Correlation Analysis of Metabolites With Clinical Parameters

We analyzed the correlation of the abundance of 55 differential metabolites with several AS clinical parameters, including BASFI, BASDAI, ASDAS, CRP, ESR, and fat-saturation score. As shown in **Table 2**, BASFI was positively correlated with L-glutamate and tyramine (*p*<0.05). Besides, a significantly positive correlation was observed between tyramine with ASDAS (r = 0.358, *p* = 0.044) and CRP (r = 0.389, *p* = 0.028). The abundance of two glycerophospholipid metabolites, namely 1-stearoyl-sn-glycerol-3-phosphocholine and lysoPC(18:0/0:0) were negatively correlated with ESR (r = −0.394, *p* = 0.026 and r = −0.404, *p* = 0.022) and positively correlated with fat-saturation score (r = 0.329, *p* = 0.066 and r = 0.357, *p* = 0.045). These results suggested that these discrepant serum metabolites were associated with the extent of inflammation and chronic lesions.

**Table 2:** The correlation analysis of differentiated metabolites and clinical parameters among AS patients.

Metabolites	Clinical parameters	r	*p*
BASFI	L-Glutamate	0.463	0.008**
	Tyramine	0.500	0.004**
ASDAS	Tyramine	0.358	0.044**
CRP	Tyramine	0.389	0.028**
	1-Stearoyl-sn-glycerol-3-phosphocholine	−0.297	0.099*
	LysoPC(18:0/0:0)	−0.312	0.082*
ESR	Tyramine	0.337	0.060*
	1-Stearoyl-sn-glycerol-3-phosphocholine	−0.394	0.026**
	LysoPC(18:0/0:0)	−0.404	0.022**
Fat-saturated	1-Stearoyl-sn-glycerol-3-phosphocholine	0.329	0.066*
	LysoPC(18:0/0:0)	0.357	0.045**

AS, ankylosing spondylitis; BASDAI, Bath Ankylosing Spondylitis Activity Index; BASFI, Bath Ankylosing Spondylitis Functional Index; ASDAS, Ankylosing Spondylitis Disease Activity Score; CRP, C-reactive protein; ESR, erythrocyte sedimentation rate; r: correlation coefficient; *p < 0.1, **p < 0.05.

### Enrichment and Pathways Analysis

**Figure 4:**
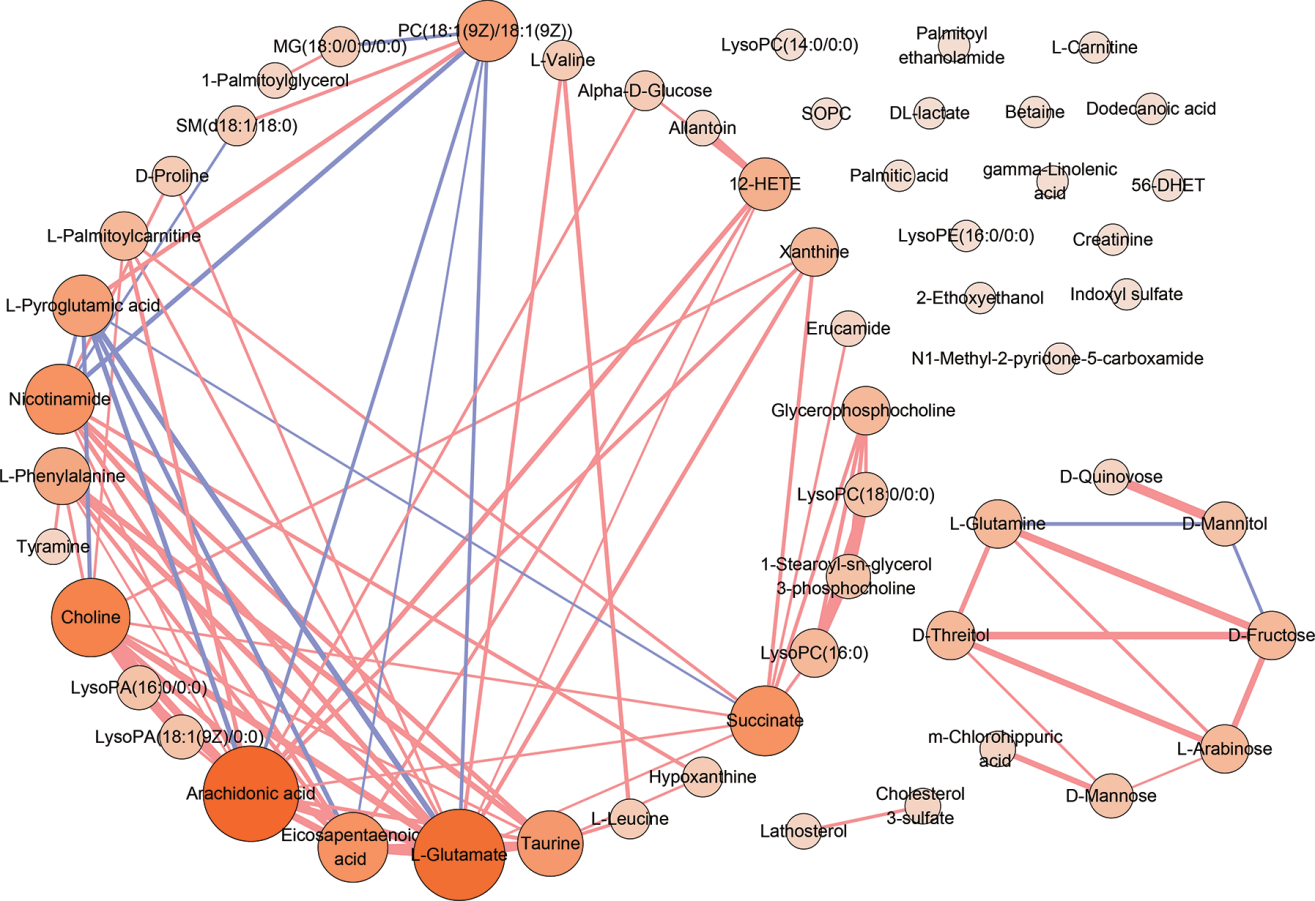
The correlation analysis of 55 serum differential metabolites. The correlation-based network deduced from 55 differential serum metabolites. Only moderate correlation or above (correlation coefficient ≥0.6 or ≤−0.6, p <0.05) serum metabolite pairs were included. Red edges denote correlation coefficient ≥0.6. Purple edges denote correlation coefficient ≤−0.6. The width of edge represents the intensity of correlation. Nodes denotes metabolites. The color depth and the sizes of nodes represent the number of correlated metabolites linked to the certain metabolite (degree).

To visualize the correlation between AS-associated metabolites, we construct a correlation-based network with the 55 differential serum metabolites. In this network, only serum metabolite pairs of moderate correlation or above (correlation coefficient ≥0.6 or ≤−0.6, p <0.05) were included (**Figure 4**). The metabolites frequently correlated with other metabolites tend to contribute to the development of the disease. According to the number of correlated compounds linked to the certain metabolite (defined as degree centrality), the most interconnected metabolites were arachidonic acid (degree=15), L-glutamate (degree=14), choline (degree=11), succinate (degree=9), nicotinamide (degree=9), eicosapentaenoic acid (degree=9), taurine (degree=8), PC(18:1(9Z)/18:1(9Z)) (degree=7), L-pyroglutamic acid (degree=7), L-phenylalanine (degree=6), 12-HETE (degree=5).

**Figure 5:**
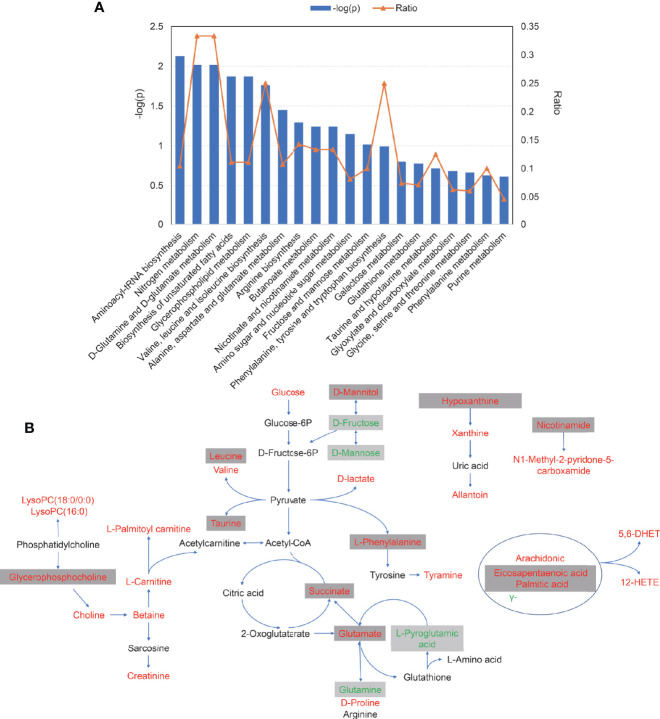
The pathways analysis of 55 serum differential metabolites. **(A)** The plot of significantly disturbed pathways using MetaboAnalyst. The x-axis displays the pathways. The y-axis displays the −log of p-value calculated by hypergeometric test. The orange dots connected by a thin line represent the ratio, which is calculated as follows: the number of differential metabolites in each pathway, divided by the total number of metabolites in the pathway. **(B)** Schematic scheme of disturbed metabolic pathways. Metabolites marked in red, green and black indicate metabolites significantly increased, decreased, unchanged or not measured in ankylosing spondylitis patients compared with control, respectively. Metabolites in grey box represent metabolites significantly altered after TNFi treatment.

Pathway enrichment analysis was performed with the serum differential metabolites data in AS. As is presented in **Figure 5A**, perturbed metabolic pathways of 55 metabolites mainly involved aminoacyl-tRNA biosynthesis, nitrogen metabolism, D-glutamine and D-glutamate metabolism, biosynthesis of unsaturated fatty acids, glycerophospholipid metabolism, valine, leucine and isoleucine biosynthesis and alanine, aspartate and glutamate metabolism. Finally, a schematic scheme of proposed metabolic pathways was presented to visualize the interaction between the differential metabolites (**Figure 5B**). The metabolites with significant changes were mapped onto several biochemical processes including amino acid biosynthesis, glycolysis, glutaminolysis, fatty acids biosynthesis, choline metabolism, and purine metabolism, which were also partly influenced by TNF inhibitors.

## Discussion

In this study, a cohort of 72 participants including 32 AS patients (pre- and post-treatment samples were collected) and 40 healthy volunteers were enrolled. We utilized a more sensitive untargeted metabolomics platform, ultra-performance LC-MS, to profile a spectrum of metabolites in the serum of AS patients and controls. This detection procedure yielded a total of 55 significantly different metabolites, including amino acids, carbohydrates, fatty acyls, glycerophospholipids, purines and purine derivatives. Much to the frustration of patients and clinicians, the diagnosis of AS is often delayed, which hinders the timely intervention, given that biologics have proven highly effective. Based on LASSO analysis, we generated a diagnostic panel comprising five metabolites (L-glutamate, arachidonic acid, L-phenylalanine, PC(18:1(9Z)/18:1(9Z)), 1-palmitoylglycerol), capable of discriminating healthy individuals from AS participants. The ROC analysis of the panel revealed a high diagnostic value for AS (AUC=0.998, 95%CI : 0.992–1.000). We propose that the combination of the 5 compounds is potentially a novel biomarker panel for the diagnosis of AS. Furthermore, treatment with TNF inhibitors can help to restore the equilibrium of the 21 metabolites. However, there is no specific metabolic phenotype that predicts outcomes following initiation of anti-TNF therapy.

As recently reviewed, it has been suggested that metabolomics analysis is a promising tool for better understanding of AS pathogenesis (14). In our study, the level of most disturbed metabolites was elevated in patients versus HCs as well as in pre-treatment versus post-treatment. The results appeared to imply the active biosynthetic demands in the disease, which relieves after TNF inhibitors treatment. As the most prominent discriminatory metabolites, most amino acids were up-regulated in serum from AS subjects, which was consistent with the previous findings (15). L-Glutamate, a metabolite in the LASSO-based model, was one of the most correlated metabolites in the network (degree=14). Besides, pathway analysis also indicated the disease was highly correlated to D-glutamine and D-glutamate metabolism. It could be inferred that glutaminase 1 is over-activated in AS, the first enzyme in glutaminolysis which converts glutamine to glutamate, from the increased level of glutamate and decreased level of glutamine in AS. Glutaminolysis is considered to be the main source of energy production in tumor cells (16) and also a well-known source of energy for effector T cells and facilitates Th17 development (17). Previous research reported that glutaminolysis played a key role in the cell growth of fibroblast-like synoviocytes in rheumatoid arthritis (RA) (18). This finding could also account for the tumor-like metabolic characteristic of AS to some extent. The specific mechanism of glutaminolysis in AS was never reported and warrants future studies. In line with the prior report (15), L-phenylalanine, a significant biomarker in the diagnostic model, was up-regulated in patients. Tyramine, a product of tyrosine (**Figure 5B**), was also increased in AS and positively correlated with BASFI, ASDAS, CRP and ESR. Tyrosine and phenylalanine are precursors for catecholamines including tyramine, dopamine, epinephrine, and norepinephrine. Therefore, we speculated that the catecholamine system in AS may be active. Two branched-chain amino acids (BCAAs) (leucine and valine) were elevated in patients and did not change with the treatment. Noteworthy, the findings were in line with the recent literature (19). BCAAs were observed to increase in multiple chronic diseases and recognized as the biomarkers and causal agents of cardiometabolic diseases (20).

Several unsaturated fatty acids (including eicosapentaenoic acid, γ-linolenic acid, and arachidonic acids) and one saturated fatty acid (palmitic acid) were altered in AS. The perturbed fatty acid metabolism was also observed in previous literature (21). Arachidonic acid was a hub metabolite in the correlation network and the diagnostic model, reported to present a positive correlation with BASDAI (22). These fatty acids could mediate inflammation, platelet aggregation and immune function either directly or upon its conversion to eicosanoids (prostaglandins, thromboxanes, and leukotrienes) (23).

Intriguingly, evidence of altered carbohydrate metabolism in patients with AS was also observed, with higher levels of glucose, D-lactate, D-mannitol, and succinate along with lower levels of D-mannose and D-fructose metabolites in patients. As the anaerobic oxidation products of glucose, the elevated D-lactate reflected the oxygen shortage in AS patients. The increased levels of glucose and succinate indicated the patients could not utilize glucose effectively, resulting in ATP insufficiency.

Notably, choline metabolism was significantly perturbed. The concentration of choline, betaine as well as multiple phospholipids were increased in AS. Choline is a basic constituent of lecithin (namely phosphatidylcholine) and can be oxidized to form betaine, which is a methyl source for many reactions. The elevated choline was also observed in the fecal samples of AS and IBD patients, which was associated with the level of intestinal inflammation (24, 25). Two kinds of phosphatidylcholine, lysoPC(18:0/0:0) and lysoPC(16:0) were increased, which stood in contrast to the previous findings (15). We observed an inverse correlation between serum lysoPC(18:0/0:0) and inflammatory markers (CRP and ESR). The discrepancy might result from the different status of disease activity of studies. Altogether, the choline might play a role in the inflammation and required further validation.

Some limitations should be noted in our study. The sample size was insufficient, hindering subsequent subgroup analysis. It was a limitation that we did not perform an independent cohort validation. Further research concerning the mechanism was also required to understand the effect of these metabolites in AS. We are just beginning to understand the functional implications of these alterations, with much more work to be done exploring the potential clinical significance and therapeutic implications of abnormalities of important metabolites. Considering that the nature of our study is a case-control association study, we could draw no definitive causal conclusions and the results might be open to interpretation. This was not the first study describing metabolic signature in AS patients. However, to our knowledge, this is the first study reporting the metabolomics signatures associated with TNF inhibitors treatment in AS patients. Although no metabolites were identified to predict the response to TNF inhibitors, the similar metabolic status between the sensitive and resistant groups may suggest the baseline metabolic status is insufficient to predict outcomes following anti-TNF therapy. The strength of this study is that all AS patients were disease-active and naïve to the treatment of TNF inhibitors at the time of enrollment. Apart from that, we performed a correlation analysis of metabolites with clinical parameters. These analyses gave clinical significance to our findings. Furthermore, a network with differential metabolites was beneficial to understanding the pathogenesis of AS.

In summary, this study characterized the serum metabolomic pattern of AS. We developed a five-metabolites-based panel that could distinguish AS patients from healthy controls. The panel could serve as a promising diagnostic tool and a complement test for detection of AS. This study has also yielded new knowledge about the pathogenesis of the disease and the systemic effects of TNF inhibitors. Therefore, further research regarding the disturbed metabolic pathway will provide new strategies for the treatment of AS.

## Data Availability Statement

The raw data supporting the conclusions of this article will be made available by the authors, without undue reservation.

## Ethics Statement

The studies involving human participants were reviewed and approved by the Ethics Committee of the Third Affiliated Hospital, Sun Yat-Sen University. The patients/participants provided their written informed consent to participate in this study.

## Author Contributions

All authors contributed to the article and approved the submitted version. JG had full access to all of the data in the study and takes responsibility for the integrity of the data and the accuracy of the data analysis. Study conception and design: JO, MX, JG. Acquisition of data: JO, MX, YH, LT, ZC, SC, QW, JG. Analysis and interpretation of data: JO, MX, JG.

## Funding

This work was supported by the National Natural Sciences Foundation of China Grant (grant number 81571595, 81871294); Science and Technology Planning Project of Guangdong Province (grant number 2016A020216013) and Major program of Health Medical Collaborate Innovation of Guangzhou (grant number 201604020013).

## Conflict of Interest

The authors declare that the research was conducted in the absence of any commercial or financial relationships that could be construed as a potential conflict of interest.
