# Supplementary material for: Serum Metabolomics Signatures Associated With Ankylosing Spondylitis and TNF Inhibitor Therapy
Source: Front Immunol. 2021 Feb 19;12:630791. doi: 10.3389/fimmu.2021.630791 (PMC7933516; doi:10.3389/fimmu.2021.630791)
Supplement: Supplementary file 1 [file DataSheet_1.docx]

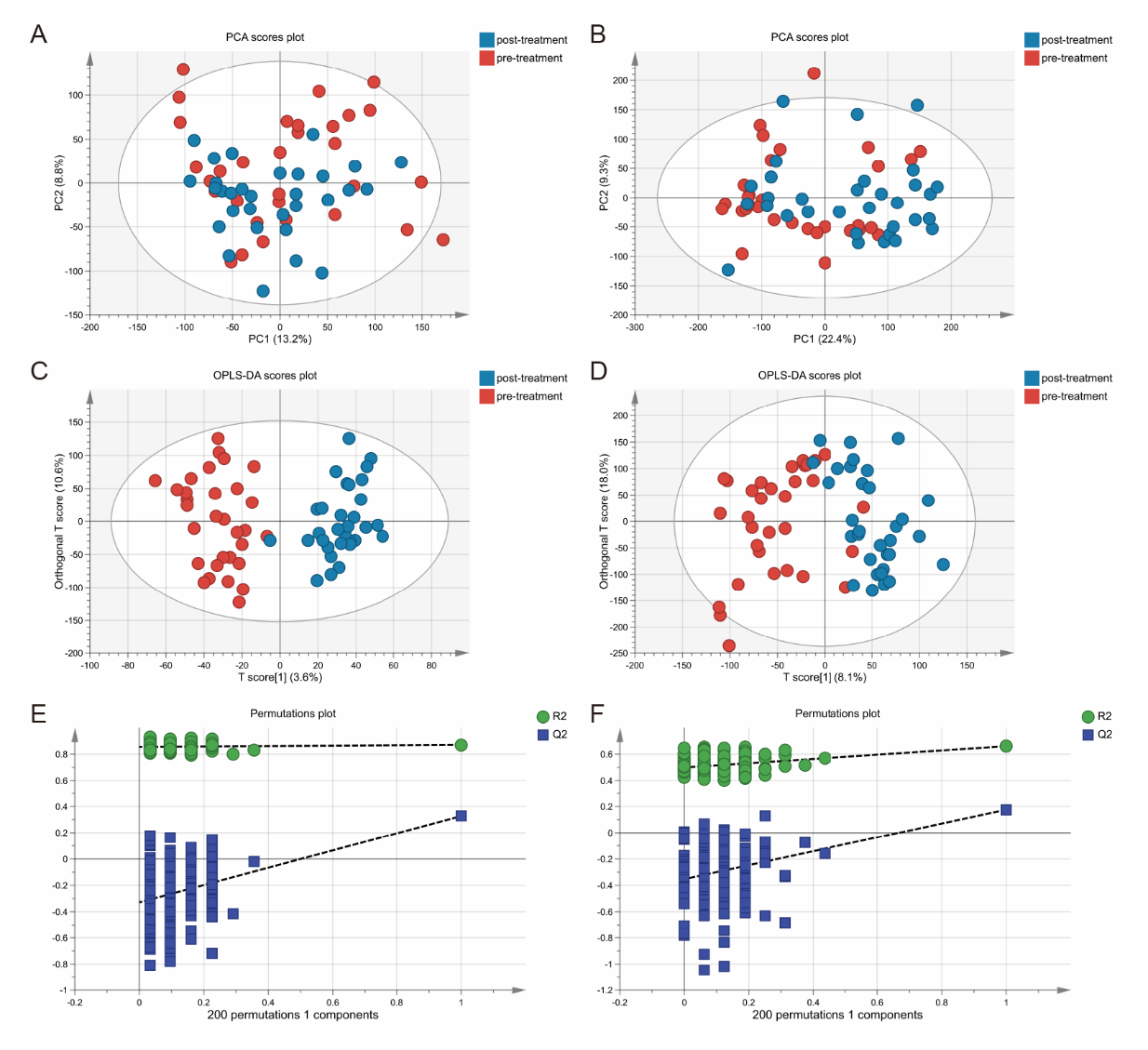


Figure S1. Multivariate statistical analysis of serum metabolites in pre-treatment and post-treatment ankylosing spondylitis patients.

(A, B) The scatter plots of principal component analysis (PCA) were based on the serum metabolic data in positive and negative ion mode. Pre-treatment and post-treatment patients were denoted with red and blue circles respectively. (C, D) Orthogonal partial least-squares discriminant analysis (OPLS-DA) scatter plots were based on the serum metabolic profiles in positive and negative ion mode. (E, F) The statistical validation of the corresponding OPLS-DA models by permutation tests (200 times).


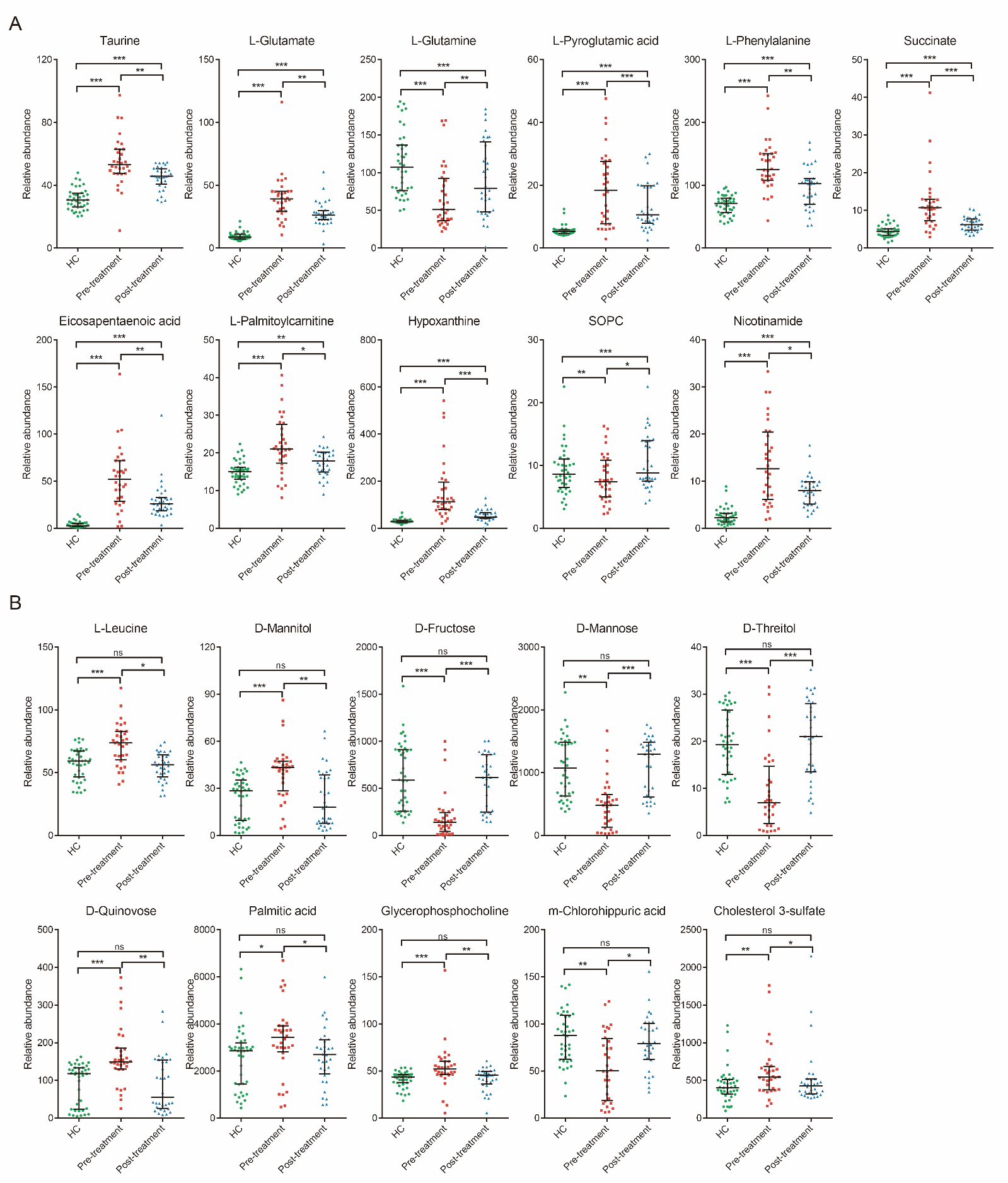


Figure S2. The scatter plot of relative abundance (median with interquartile range) of 21 metabolites in healthy controls (HCs, green dots), pre-treatment (red dots) and post-treatment (blue dots) ankylosing spondylitis (AS) patients. ns *p* >0.05; * *p* <0.1, ** *p* <0.05, *** *p* <0.01.
