# Supplementary material for: Serum Metabolomics Signatures Associated With Ankylosing Spondylitis and TNF Inhibitor Therapy
Source: Front Immunol. 2021 Feb 19;12:630791. doi: 10.3389/fimmu.2021.630791 (PMC7933516; doi:10.3389/fimmu.2021.630791)
Supplement: Supplementary file 2 [file DataSheet_2.docx]

**Supplementary Methods**

- 1. **Sample collection and preparation**

Blood samples acquired from AS patients and HCs were obtained with no additive vacuum blood collection tubes after overnight fasting. Serum was separated after standing at room temperature for 2 hours and centrifuging at 3000r/min for 5 minutes. The serum samples were stored at -80 °C before further analysis. Before the LC-MS analysis, the samples were thawed at room temperature. All samples were extracted and precipitated protein with 300μL methanol, then added with 25μL internal standard solution. After vortexing for 30 seconds and centrifuging at 12000 rpm for 15 minutes under 4 °C, 200μL of supernatant was transferred into a vial for analysis. To evaluate the repeatability and reliability of the analytical conditions, the quality control samples (QC) were prepared by mixing up the equal volume of each sample and then processed following the same procedure.

- 1. **LC–MS metabolomic profiling**

Samples were analyzed by liquid chromatography platform (1290 Infinity LC, Agilent Technologies) coupled to a quadrupole time-of-flight mass spectrometry (AB Sciex TripleTOF 6600). Separation was achieved on a 2.1 mm × 100 mm ACQUIY UPLC BEH 1.7 µm column (waters, Ireland) with a programmed gradient elution of (A) 25 mM ammonium acetate and 25 mM ammonium hydroxide in water and (B) acetonitrile (0- 1.0 min, 85% B; 1.0- 12.0 min, 85%-65% B; 12.0-12.1 min, 65%-40% B; 12.1- 15.0 min, 40% B; 15.0-15.1 min, 40%-85% B; 15.1-20.0 min, 85% B). The flow rate was 0.30 ml/min and the temperature of autosampler was maintained at 4 °C.

In MS only acquisition, data were acquired between m/z 60-1,000 Da and the accumulation time for TOF MS scan was set at 200 ms per spectrum. In auto MS/MS acquisition, data were acquired between m/z 25 and 1,000 Da and the accumulation time for product ion scan was set at 50 ms per spectrum. The electrospray ionization source (ESI) source conditions were set as follows: Ion Source Gas1 (Gas1) as 60, Ion Source Gas2 (Gas2) as 60, curtain gas (CUR) as 30, source temperature: 600℃, IonSpray Voltage Floating (ISVF) ± 5500 V. The product ion scan was acquired using information-dependent acquisition (IDA) with high sensitivity mode selected. The parameters were set as follows: the collision energy (CE) was fixed at 35 V with ± 15 eV; declustering potential (DP), 60 V (+) and −60 V (−); exclude isotopes within 4 Da, candidate ions to monitor per cycle: 10.

- 1. **Data Processing and statistical analysis**

The raw data were converted to MzXML files using ProteoWizard. XCMS software was used for peak detection and alignment. The data processing procedures comprised filtering, imputation of missing values (R package “DMwR”) and area normalization. Before data analysis, the relative standard deviation (RSD) of each feature in QC samples was calculated, and features with RSD greater than 30% were excluded. Then the data matrix was imported into SIMCA-P software (version 14.1, Umetrics AB, Umea, Sweden) for multivariate statistical analysis including principal component analysis (PCA) and orthogonal partial least-squares discriminant analysis (OPLS-DA). PCA is an unsupervised clustering algorithm which provides an overview of metabolites. The cross-validation through permutation tests were conducted to evaluate the robustness of the models. The variable importance in the projection (VIP) value of each variable in the OPLS-DA model was calculated to indicate its contribution to the classification. Univariate Wilcoxon rank-sum test was applied to metabolites with VIP values >1.0. The false discovered rate (FDR) was used to adjust *p* values. The differential metabolites that satisfied the criterion of VIP values of >1.0 and FDR of <0.05 were considered as biomarker candidates. The significant remaining features were identified through database searches including the Human Metabolome Database (<http://www.hmdb.ca>), METLIN (<https://metlin.scripps.edu>), and Chemspider (<http://www.chemspider.com/>). The diagnostic efficacy of each metabolite was assessed by receiver operating characteristic curve (ROC). The metabolites with the area under the curves (AUCs) greater than 0.75 were selected to construct a diagnostic model with Least absolute shrinkage and selection operator (LASSO) regression (R package “glmnetcr”). The enrichment and pathways analysis were performed with MetaboAnalyst 4.0 and Kyoto Encyclopedia of Genes and Genomes (KEGG) pathway database. The visualization of metabolites network was obtained with the MetScape app in Cytoscape software (version 3.6.0). The results were presented as mean ± standard deviation (SD) for continuous variables and as percentage for categorical variables. GraphPad Prism (version 6.02, GraphPad Software, San Diego, CA, USA) was used for statistical analysis of the data. *P* < 0.05 was considered statistically significant.
