# Supplementary material for: Serum Metabolomics Signatures Associated With Ankylosing Spondylitis and TNF Inhibitor Therapy
Source: Front Immunol. 2021 Feb 19;12:630791. doi: 10.3389/fimmu.2021.630791 (PMC7933516; doi:10.3389/fimmu.2021.630791)
Supplement: Supplementary file 3 [file Table_1.docx]

Table S1 Significantly increased serum metabolites of AS patients compared with HC in discovery stage.

| Metabolites  (n=41) | HMDB ID | change in AS (vs HC) | | |  | change in post-treatment (vs pre-treatment) | | |
| --- | --- | --- | --- | --- | --- | --- | --- | --- |
|  |  | VIP-value | *p^a^* | FC |  | VIP-value | *p^a^* | FC |
| Taurine | HMDB0000251 | 2.43 | 2.56E-13 | 1.77 |  | 3.05 | 1.04E-03 | 0.81 |
| L-Glutamate | HMDB0000148 | 2.90 | 5.74E-18 | 4.17 |  | 3.38 | 1.51E-03 | 0.69 |
| L-Phenylalanine | HMDB0000159 | 3.97 | 2.56E-13 | 1.90 |  | 5.24 | 1.04E-03 | 0.73 |
| L-Leucine | HMDB0000687 | 1.50 | 9.17E-05 | 1.27 |  | 2.07 | 1.24E-02 | 0.85 |
| D-Proline | HMDB0003411 | 1.05 | 1.48E-12 | 1.75 |  | - | - | - |
| L-Valine | HMDB0000883 | 1.68 | 1.36E-08 | 1.32 |  | - | - | - |
| Tyramine | HMDB0000306 | 1.48 | 6.53E-04 | 1.37 |  | - | - | - |
| Choline | HMDB0000097 | 9.98 | 1.66E-13 | 3.00 |  | - | - | - |
| Betaine | HMDB0000043 | 4.10 | 1.38E-04 | 1.24 |  | - | - | - |
| L-Carnitine | HMDB0000062 | 1.32 | 1.38E-04 | 1.18 |  | - | - | - |
| Alpha-D-Glucose | HMDB0003345 | 1.37 | 2.53E-03 | 1.60 |  | - | - | - |
| D-lactate | HMDB0001311 | 7.46 | 8.15E-03 | 1.33 |  | - | - | - |
| Succinate | HMDB0000254 | 1.42 | 2.80E-09 | 2.65 |  | 1.07 | 8.73E-05 | 0.53 |
| D-Mannitol | HMDB0000765 | 1.66 | 4.65E-05 | 1.69 |  | 1.71 | 1.91E-03 | 0.58 |
| D-Quinovose | HMDB0062477 | 3.50 | 1.26E-05 | 1.80 |  | 3.58 | 2.63E-03 | 0.55 |
| Eicosapentaenoic acid | HMDB0001999 | 1.87 | 3.74E-16 | 4.93 |  | 5.17 | 1.64E-03 | 0.56 |
| Palmitic acid | HMDB0000220 | 7.86 | 1.44E-02 | 1.30 |  | 7.46 | 4.18E-02 | 0.79 |
| Arachidonic acid | HMDB0001043 | 1.31 | 1.20E-14 | 5.48 |  | - | - | - |
| 12-HETE | HMDB0062287 | 4.48 | 1.35E-09 | 8.87 |  | - | - | - |
| 5,6-DHET | HMDB0002343 | 1.03 | 6.65E-03 | 1.96 |  | - | - | - |
| 1-Palmitoylglycerol | HMDB0031074 | 3.66 | 8.84E-09 | 1.70 |  | - | - | - |
| Erucamide | NA | 1.78 | 3.23E-06 | 1.64 |  | - | - | - |
| MG(18:0/0:0/0:0) | HMDB0011131 | 3.26 | 2.50E-05 | 1.94 |  | - | - | - |
| L-Palmitoyl carnitine | HMDB0000222 | 1.18 | 2.50E-06 | 1.49 |  | 2.04 | 1.22E-02 | 0.79 |
| Hypoxanthine | HMDB0000157 | 5.10 | 5.86E-14 | 5.32 |  | 12.38 | 3.96E-05 | 0.34 |
| Xanthine | HMDB0000292 | 1.02 | 2.42E-05 | 1.79 |  | - | - | - |
| Allantoin | HMDB0000462 | 1.03 | 3.61E-02 | 2.39 |  | - | - | - |
| LysoPC(18:0/0:0) | HMDB0010384 | 3.14 | 3.27E-02 | 1.12 |  | - | - | - |
| LysoPE(16:0/0:0) | HMDB0011503 | 1.04 | 2.61E-04 | 1.34 |  | - | - | - |
| 1-Stearoyl-sn-glycerol-3-phosphocholine | NA | 1.31 | 3.46E-02 | 1.12 |  | - | - | - |
| Glycerophosphocholine | HMDB0000086 | 1.10 | 1.67E-05 | 1.30 |  | 2.29 | 4.35E-03 | 0.78 |
| LysoPC(16:0) | HMDB0010382 | 6.79 | 1.39E-02 | 1.16 |  | - | - | - |
| LysoPC(14:0/0:0) | HMDB0010379 | 1.19 | 7.80E-04 | 1.42 |  | - | - | - |
| LysoPA(18:1(9Z)/0:0) | HMDB0007855 | 1.11 | 2.74E-06 | 3.46 |  | - | - | - |
| LysoPA(16:0/0:0) | HMDB0007853 | 1.43 | 9.81E-04 | 3.83 |  | - | - | - |
| Nicotinamide | HMDB0001406 | 1.67 | 5.86E-13 | 5.31 |  | 2.95 | 2.54E-02 | 0.58 |
| N1-Methyl-2-pyridone-5-carboxamide | HMDB0004193 | 1.68 | 5.47E-04 | 2.09 |  | - | - | - |
| Lathosterol | HMDB0001170 | 1.10 | 1.30E-03 | 1.39 |  | - | - | - |
| Cholesterol 3-sulfate | HMDB0000653 | 4.58 | 8.15E-03 | 1.42 |  | 3.30 | 4.18E-02 | 0.84 |
| 2-Ethoxyethanol | HMDB0031213 | 1.25 | 1.19E-04 | 1.71 |  | - | - | - |
| Palmitoyl ethanolamide | HMDB0002100 | 1.68 | 2.74E-04 | 1.17 |  | - | - | - |

HC, health control; AS, ankylosing spondylitis; VIP, variable importance in projection; FC, fold change. NA, data was not available. ^a^ *p* value was adjusted after controlling the false discovery rate.

Table S2 Significantly decreased serum metabolites of AS patients comparing to HC in discovery stage.

| Metabolites  (n=14) | HMDB ID | change in AS (vs HC) | | |  | change in post-treatment (vs pre-treatment) | | |
| --- | --- | --- | --- | --- | --- | --- | --- | --- |
|  |  | VIP-value | *p^a^* | FC |  | VIP-value | *p^a^* | FC |
| Creatinine | HMDB0000562 | 2.28 | 6.40E-03 | 0.85 |  | - | - | - |
| L-Pyroglutamic acid | HMDB0000267 | 1.08 | 3.40E-13 | 0.37 |  | 1.20 | 9.15E-04 | 1.60 |
| L-Glutamine | HMDB0000641 | 2.90 | 2.97E-05 | 0.60 |  | 1.18 | 5.43E-03 | 1.69 |
| D-Mannose | HMDB0000169 | 1.85 | 1.74E-03 | 0.69 |  | 12.49 | 4.73E-05 | 2.27 |
| D-Fructose | HMDB0000660 | 10.78 | 5.43E-08 | 0.32 |  | 9.65 | 3.63E-05 | 2.81 |
| D-Threitol | HMDB0004136 | 1.72 | 2.97E-06 | 0.48 |  | 1.66 | 7.97E-05 | 2.15 |
| L-Arabinose | HMDB0000646 | 1.14 | 2.97E-06 | 0.49 |  | - | - | - |
| γ-Linolenic acid | HMDB0003073 | 2.24 | 7.62E-03 | 0.81 |  | - | - | - |
| Dodecanoic acid | HMDB0000638 | 1.77 | 1.22E-02 | 0.59 |  | - | - | - |
| PC(18:1(9Z)/18:1(9Z)) | HMDB0062690 | 1.78 | 1.96E-16 | 0.40 |  | - | - | - |
| SOPC | NA | 1.77 | 2.50E-03 | 0.51 |  | 1.58 | 3.77E-02 | 1.33 |
| SM(d18:1/18:0) | HMDB0001348 | 1.40 | 1.93E-13 | 0.33 |  | - | - | - |
| m-Chlorohippuric acid | HMDB0001309 | 2.85 | 1.27E-03 | 0.64 |  | 2.23 | 1.81E-02 | 1.46 |
| Indoxyl sulfate | HMDB0000682 | 7.74 | 3.70E-02 | 0.69 |  | - | - | - |

HC, health control; AS, ankylosing spondylitis; VIP, variable importance in projection; FC, fold change. NA, data was not available. ^a^ *p* value was adjusted after controlling the false discovery rate.
